# Neighborhood Characteristics, Functional Limitations, and Falls Among Older Adults

**DOI:** 10.1093/geroni/igaf122.2151

**Published:** 2025-12-31

**Authors:** Jeein Law (Jang, Debasree Das Gupta, Uma Kelekar

**Affiliations:** University of Massachusetts Boston, Boston, Massachusetts, United States; Utah State University, Logan, Utah, United States; Marymount University, Arlington, Virginia, United States

## Abstract

Falls among older adults (≥65 years) pose a significant public health concern, leading to increased morbidity, loss of independence, and substantial healthcare costs. While prior research has largely focused on individual health factors, less is known about how perceived neighborhood environment influences fall risk, particularly among older adults with functional limitations. We examined whether the relationship between neighborhood environment and falls was moderated by functional limitations. Using data from the Health and Retirement Study, we analyzed two waves (2014-2016 baseline and 2016-2018 follow-up) (n = 2,486). Perceived neighborhood social cohesion and physical disorder were assessed at baseline. Functional limitations were classified into three categories (no/mild/severe) based on difficulties in daily and instrumental activities of daily living and Nagi strength and mobility tasks. Falls were self-reported at follow-up. Survey-weighted logistic regression models estimated the associations between neighborhood characteristics and falls, controlling for sociodemographic and health-related factors. Interaction terms examined whether the effects of neighborhood characteristics varied by functional status. Results showed perceived physical disorder but not perceived social cohesion was significantly associated with fall risk. However, higher levels of physical disorder were associated with decreased fall risk among older adults with mild and severe functional limitations, which may be indicative of reduced mobility or outdoor engagement of these groups when exposed to higher levels of disorder. These findings underscore the importance of neighborhood context in shaping fall risk, particularly among older adults with functional limitations. Policymakers and urban planners should prioritize neighborhood enhancements, such as maintaining clean, hazard-free public spaces for healthy aging.

